# An IQSEC2 Mutation Associated With Intellectual Disability and Autism Results in Decreased Surface AMPA Receptors

**DOI:** 10.3389/fnmol.2019.00043

**Published:** 2019-02-20

**Authors:** Eli J. Rogers, Reem Jada, Kinneret Schragenheim-Rozales, Megha Sah, Marisol Cortes, Matthew Florence, Nina S. Levy, Rachel Moss, Randall S. Walikonis, Raz Palty, Reut Shalgi, Daniela Lichtman, Alexandra Kavushansky, Nashaat Z. Gerges, Itamar Kahn, George K. E. Umanah, Andrew P. Levy

**Affiliations:** ^1^Technion Faculty of Medicine, Technion Israel Institute of Technology, Haifa, Israel; ^2^Department of Physiology and Neurobiology, University of Connecticut, Storrs, CT, United States; ^3^Department of Neurology, Johns Hopkins University, Baltimore, MD, United States; ^4^Department of Biopharmaceutical Sciences and Department of Cell Biology, Neurobiology and Anatomy, Medical College of Wisconsin, Milwaukee, WI, United States

**Keywords:** IQSEC2, Arf6, GEF, AMPA, calmodulin, IQ domain, intellectual disability, autism

## Abstract

We have recently described an A350V mutation in IQSEC2 associated with intellectual disability, autism and epilepsy. We sought to understand the molecular pathophysiology of this mutation with the goal of developing targets for drug intervention. We demonstrate here that the A350V mutation results in interference with the binding of apocalmodulin to the IQ domain of IQSEC2. We further demonstrate that this mutation results in constitutive activation of the guanine nucleotide exchange factor (GEF) activity of IQSEC2 resulting in increased production of the active form of Arf6. In a CRISPR generated mouse model of the A350V IQSEC2 mutation, we demonstrate that the surface expression of GluA2 AMPA receptors in mouse hippocampal tissue was significantly reduced in A350V IQSEC2 mutant mice compared to wild type IQSEC2 mice and that there is a significant reduction in basal synaptic transmission in the hippocampus of A350V IQSEC2 mice compared to wild type IQSEC2 mice. Finally, the A350V IQSEC2 mice demonstrated increased activity, abnormal social behavior and learning as compared to wild type IQSEC2 mice. These findings suggest a model of how the A350V mutation in IQSEC2 may mediate disease with implications for targets for drug therapy. These studies provide a paradigm for a personalized approach to precision therapy for a disease that heretofore has no therapy.

## Introduction

IQSEC2 is an X-linked gene which has been previously associated with intellectual disability (ID), autism and epilepsy (Shoubridge et al., 2010, 2019; Fieremans et al., 2015; Alexander-Bloch et al., 2016; Kalscheuer et al., 2016; Zerem et al., 2016; Mignot and Depienne, 2018) with mutations in IQSEC2 accounting for approximately 2% of patients with ID and epilepsy referred for exome sequencing (Heyne et al., 2018). Understanding the molecular pathophysiology of IQSEC2 mutations may allow for a personalized treatment program to provide much-needed hope and help to affected children and their families.

The IQSEC2 protein is localized in excitatory synapses as part of the NMDA receptor complex via interaction with post-synaptic density proteins DLG1, DLG2, and DLG4 and has been proposed to play a role in synaptic plasticity and dendritic spine formation (Murphy et al., 2006; Sakagami et al., 2008; Hinze et al., 2017). Biochemically IQSEC2 is a member of the GEF (guanine nucleotide exchange factor) family of proteins whose role is to promote exchange of GDP for GTP on specific Arfs (ADP ribosylation factors) and thereby activate the Arf. The target Arf for IQSEC2 is not known but binding of IQSEC2 to Arf6 has been demonstrated *in vitro* (Sakagami et al., 2008). Arf6, similar to other Arfs, regulates actin dynamics in dendritic spines and membrane trafficking, and is the only Arf which regulates trafficking between the cell surface membrane and endocytotic membranes (Donaldson, 2003; Choi et al., 2006; Jaworski, 2007). The GEF activity of IQSEC2, mediated through ARF6, has recently been demonstrated to be required for the activity dependent removal of α-amino-3-hydroxyl-5-methyl-4-isoxazolepropionic acid (AMPA) receptors (Brown et al., 2016; Petersen et al., 2018) from the surface of hippocampal neurons. The regulation of surface synaptic AMPA receptors has been shown to be critically involved in learning and memory processes with alterations in AMPA trafficking being associated with cognitive impairment and social behavioral abnormalities (Awasthi et al., 2018; Medin et al., 2018; Parkinson and Hanley, 2018). Demonstration that IQSEC2 can regulate AMPA trafficking (Brown et al., 2016) may therefore provide a mechanistic link for the severe intellectual disability and abnormalities in social behavior associated with mutations in IQSEC2.

The IQSEC2 gene contains 15 exons and codes for a protein of 1488 amino acids (long isoform) with 98.5% homology between murine IQSEC2 and human IQSEC2. The coding sequence contains several canonical domains notably a catalytic domain (SEC7) [aa 746–939] characteristic of all GEFs promoting GTP exchange and an IQ like domain [aa 347–376] which has been suggested to bind calmodulin and thereby modulate the GEF activity of IQSEC2 (Shoubridge et al., 2010).

At least 70 different mutations have been described in the IQSEC2 gene all associated with moderate to severe intellectual disability, with variable seizures and autistic traits (Shoubridge et al., 2019). The genotype-phenotype relationship for these mutations is not understood. Many of these mutations cluster in recognized functional domains of IQSEC2 such as the Sec7 and IQ domains thereby providing a possible mechanism by which they produce disease (Mignot and Depienne, 2018; Shoubridge et al., 2019). There have been no reports in animal models on how altered IQSEC2 function for any of these mutations may influence cognition or social behavior.

We have recently reported on the ID and associated disorders in a child resulting from a *de novo* mutation identified by exome sequencing in the IQSEC2 gene (A350V, i.e., valine for alanine substitution in amino acid residue 350) (Zipper et al., 2017). In this study we set out to characterize the molecular mechanisms underlying the pathophysiology of the A350V IQSEC2 mutation *in vitro* and in a CRISPR murine model with the goal of developing precise therapies to alleviate at least in part the severe clinical syndrome associated with the mutation. First, as the A350V mutation is in the IQ calmodulin binding domain of IQSEC2 we set out to define how this mutation may affect the interaction of IQSEC2 with calmodulin. Second, as other mutations in the IQ domain have been associated with changes in the ability of IQSEC2 to promote GTP exchange on Arf6 in response to calcium (Shoubridge et al., 2010; Myers et al., 2012) we investigated whether the A350V mutation may also alter Arf6 activity and whether this regulation was sensitive to calcium. Third, as IQSEC2 induced activation of Arf6 has been shown to modulate AMPA receptor trafficking (Brown et al., 2016) we sought to determine how the A350V mutation may affect this trafficking in our CRISPR model and specifically surface AMPA receptors which have been linked to learning and memory (Parkinson and Hanley, 2018). Fourth, we set out to determine whether the A350V mutation may affect basal hippocampal synaptic transmission. Finally, in an attempt to recapitulate the clinical phenotype in the CRISPR model we have assessed the effects of the A350V IQSEC2 mutation on behavioral phenotypes focusing on tests assessing locomotion, social interactions and learning.

## Materials and Methods

### DNA Constructs Used in This Study

**Figure 1:**
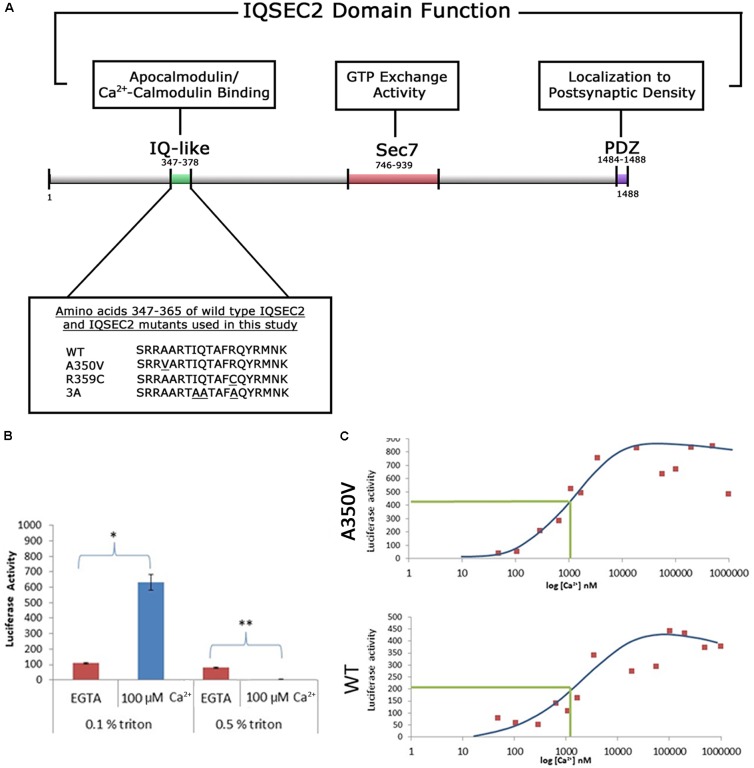
**(A)** Functional domain organization of the IQSEC2 gene and amino acid sequence of IQSEC2 IQ motif mutants used in this study. IQ-like, IQ homology motif thought to be site for interaction of IQSEC2 with calmodulin (Shoubridge et al., 2010). SEC7, catalytic domain responsible for the GEF activity of IQSEC2 (Shoubridge et al., 2010). PDZ, domain reported to mediate interaction of IQSEC2 with the post-synaptic protein PSD-95 thereby localizing IQSEC2 to the post-synaptic density (Brown et al., 2016). Inset demonstrates the amino acid sequence within the IQSEC2 IQ domain of wild type IQSEC2 and sequence variants in this region, including A350V, used in this study. Underlined residues indicate site of sequence variance from the wild type. **(B)** The effect of calcium on the binding of IQSEC2 to calmodulin depends on the triton concentration in the cell extracts. Luciferase activity of the CM sepharose beads was used as a readout of the calmodulin IQSEC2-luciferase interaction. 293T cell extracts containing wild type IQSEC2 were prepared in low (0.1%) or high (0.5%) triton and used for binding studies with calmodulin sepharose as described in methods. In the presence of low triton concentration in the extracts, calcium resulted in a significant increase in the interaction between calmodulin and IQSEC2 (indicated by ^∗^, unpaired *t*-test, *p* < 0.001, *n* = 6), while in the presence of high tritron calcium resulted in a significant decrease in the interaction between calmodulin and IQSEC2 (indicated by ^∗∗^, unpaired *t*-test, *p* < 0.001, *n* = 6). **(C)** The binding of calmodulin to wild type and A350V IQSEC2 is increased by calcium. Cell extracts containing wild type or A350V IQSEC2 and binding studies between IQSEC2 and calmodulin sepharose under defined calcium concentrations were performed as described in methods. As shown for a representative experiment for both wild type and A350V IQSEC2, calcium resulted in an increase in binding of calmodulin to IQSEC2 with a similar calcium dose-response relationship [half-maximal binding (indicated by green line) of calcium-calmodulin for IQSEC2 at a free calcium concentration of approximately 1 μM for both wild type and A350V IQSEC2]. The binding of calcium calmodulin was significantly increased for A350V IQSEC2 as compared to wild type IQSEC2 at all calcium concentrations greater than 200 nM (A350V 594 ± 59.6 vs. wild type 271 ± 41 luciferase units, *n* = 11, unpaired *t*-test, *p* < 0.0005).

The IQSEC2 wild type gene was cloned 3′ to renilla luciferase and three copies of the HA tag in pcDNA3.1 Zeo (Genscript) or 3′ to a FLAG tag in pCAGGS. The pcDNA3.1 construct expresses full length (1488aa) human IQSEC2 with an N-terminal renilla luciferase and HAx3 tag under the control of a CMV promoter, and also contains a zeocin (Zeo) gene allowing for selection of stable transformants expressing the IQSEC2 gene. Specific mutations were introduced into the renilla luciferase-wild type (WT) IQSEC2 vector or the FLAG wild type IQSEC2 vector for the studies described herein (GenScript) (Figure 1A). For production of the A350V mutation we changed the corresponding codon for IQSEC2 amino acid residue 350 from GCT (Alanine) to GTT (Valine). We also generated two additional mutant constructs in the IQ domain of IQSEC2: (1) a previously described IQSEC2 R359C mutation associated with ID (Shoubridge et al., 2010) and (2) a previously described engineered mutation containing three alanine substitutions in the IQ region at amino acid residues 354, 355 and 359 (herein called 3A) (Myers et al., 2012). All IQSEC2 constructs were verified by DNA sequencing. The genes for calmodulin (human Calm1 (NM_006888), Calm2 (NM_001743) and Calm3 (NM_ 005184) were obtained from a human ORFeome library (Yang et al., 2011) and subcloned into pcDNA3 to have a C-terminal triple FLAG tag.

### Cell Culture and Stable Cell Lines Expressing IQSEC2

HEK293T cells were propagated in DMEM with low glucose and 10% fetal calf serum (FCS). Stable cell lines (expressing either wild type or mutant A350V IQSEC2) were produced in 293T cells using selection with Zeo (200 μg/ml) after transfection with calcium phosphate.

### Arf6 Activation Assay

For the assessment of Arf6-GTP by ELISA, cell extracts were prepared from HEK293T cells stably expressing either wild type or A350V IQSEC2. ELISA was performed exactly according to manufacturer’s protocol (G-LISA Arf6 activation assay, Cytoskeleton Inc). The amount of Arf6-GTP was assessed using immobilized GGA peptide. Normalization was by total protein and/or luciferase as described in results.

For the assessment of Arf6-GTP using a GGA-3 pulldown assay and western blot, HEK293T cells were transfected with FLAG-tagged WT, A350V, or R359C IQSEC2 in pCAGGS vector by calcium phosphate. Twenty-four hours after transfection, the cultures were treated with 5 μM ionomycin or ethanol vehicle for 5 min, then lysed in 50 mM Tris-HCl, pH 7.5, 100 mM NaCl, 2 mM MgCl_2_, 0.2% SDS, 0.5% sodium deoxycholate, 1% Triton X-100, 10% glycerol, and 1x Halt protease inhibitor cocktail. An Arf6-GTP pull-down assay was carried out as described (Shoubridge et al., 2010). Briefly, lysates were cleared by centrifugation at 10,000 rpm for 10 min and incubated with GGA3:GST on glutathione-sepharose beads for 5 h at 4°C. The beads were washed and bound proteins were eluted and probed by immunoblot with rabbit anti-Arf6 (1:750, Cell Signaling #5740). Lysates were also probed against total Arf6 and with rabbit anti-FLAG (1:1000; Covance # PRB-132P) to detect expression of FLAG-IQSEC2. Bands were visualized with the use of a LiCor Odyssey imaging system and quantified with Image Studio Lite. Each band was normalized to the untreated sham-transfected control. The data were statistically analyzed by one-way ANOVA followed by Tukey’s Multiple Comparison Test for *post hoc* analysis.

### Assessment of Binding of IQSEC2 to Calmodulin *in vitro*

The binding of wild type and mutant IQSEC2 to calmodulin was assessed *in vitro* using calmodulin-sepharose (BioVision, Milpitas, CA, United States). Extracts from stably transfected cells were prepared in either buffer A [50 mM Tris pH 7.5; 150 mM NaCl, 10 mg/ml BSA; 5 mM EGTA and 0.1% Triton X-100] or buffer B [10 mM Tris pH 7.5; 150 mM NaCl, 5 mM EGTA, 5% glycerol, 0.5% Triton X-100]. Extracts were clarified by centrifugation at 14000 rpm at 4°C to remove insoluble debris and the amount of luciferase activity in the extract assessed using the Promega luciferase assay system and a Turner TD 20/20 luminometer. Extracts (10,000–100,000 luciferase units) were then incubated in buffer A or buffer B with or without CaCl_2_ in a total volume of 1 cc. The concentration of free calcium in the incubation conditions was calculated using the maxchelator algorithm^1^ which is based on the ionic strength, pH, temperature and dissociation constant of EGTA for calcium. The concentration of free calcium used in these studies ranged from 0.73 nM to 2 mM. 10 μl of calmodulin-sepharose was added to the incubation and mixed on a rotary apparatus for 3–4 h. The calmodulin-sepharose was washed twice with binding buffer, resuspended in 100 μl of luciferase reagent lysis buffer and 20 μl was assessed for luciferase activity.

### Assessment of Binding of IQSEC2 to Calmodulin in Cells

Assessment of an interaction between wild type and mutant renilla luciferase IQSEC2 constructs (wild type or mutants) and 3xFLAG tagged candidate interactors (calmodulin proteins Calm1 (NM_006888), Calm2 (NM_001743), and Calm3 (NM_005184) in HEK293T cells was performed using the Lumier assay (Taipale et al., 2012), with an automated robotic system. Briefly, constructs were cotransfected using polyethylenimine (PEI), in 96 well plates. Cells were lysed at 48 h in lysis/wash buffer [50 mM Hepes pH 7.9; 150 mM NaCl; 2 mM EDTA; 0.5% Triton X-100, 5% glycerol] and candidate interactors were pulled down in anti-FLAG antibody (Sigma, F1804) coated 384 well plates for 3 h at 4°C. The amount of renilla luciferase activity in anti-FLAG captured protein was used as a readout of the strength of the interaction between renilla-IQSEC2 and interactor-FLAG. Interaction scores designate renilla activity after pulldown normalized to FLAG ELISA, to account for potential variability in interactor levels. GFP-FLAG was used as a negative control interactor and its interaction strength and score were considered background levels. Each experiment was performed with 2–4 replicate wells for each pair of IQSEC2 (wild type or mutant) and each calmodulin. In addition, independent replicate experiments were performed on different days, four repeat experiments for the wild type IQSEC2 and the A350V mutant and two repeat experiments for the R359C and 3A mutants. Renilla activity was also measured in whole cell lysates (negative control cells, cotransfected with GFP-FLAG) in order to verify that the observed differences in interaction scores between wild type and mutant IQSEC2 and the calmodulins were not due to differences in cell viability or transfection efficiency.

### Generation of A350V IQSEC2 Mice by CRISPR

Mice were generated by CRISPR at Applied Stem Cells (Milpitas, CA, United States). We targeted murine IQSEC2 (NM_001005475.2) with the goal of generating an A350V mutation identical to that found in the human index case in which the codon GCT (Ala) at amino acid 350 is mutated to GTT (Val) with an additional AGG to CGT silent mutation (R349) in order to prevent the guide RNA g20 GGCAGCCCTGCGGCTCAGGA from targeting the same allele after repair. A single stranded oligonucleotide donor (ssODN) was synthesized with two homology arms flanking the GCT to GTT mutation site (5′CTGAGCT GCGCAGCCGCTCAAAGTTCTTATTCATACGGTACTGTCG AAAGGCTGTCTGGATGGTCCTGGCAACACGGCGGCTCA GGAAGGAGCCCCCATACTTCCTCTCCAGCATTTCCACCT GTCAGAGGAACAAGTTCAGAAAG3′) serving as the repair template during the process of homology directed repair (HDR). Synthesized ssODN donor, g20 gRNA transcripts and Cas9 mRNA were microinjected into the cytoplasm of C57BL/6J embryos. Identification of F0 successfully targeted mice were identified by Sanger sequencing. Germline transmitted F1s containing the mutation were used to generate the A350V colony used for all additional studies and continued breeding of the mice was done in a C57Bl/6J background. Approximately 1 kb of DNA was sequenced on both sides of the mutation with no other changes detected. Wild type (WT) and A350V IQSEC2 protein were also assessed by western blot from mouse brains and they were found to be of the same size as predicted. MRI structural analysis of both wild type and A350V mice revealed no gross differences in brain volume or gross structural differences in A350V mice. Hemizygous males, heterozygous and homozygous females were fertile and were housed in a germ-free animal facility and used for breeding and the studies described.

All studies for which the mice were used were approved by the Institutional Animal Care and Use Committees of the institutions in which they were performed (Technion Faculty of Medicine (IL0360212; IL1691117) and Medical College of Wisconsin (AUA1650).

### Flow Cytometry Analysis for Surface AMPA Receptors of Hippocampal Neurons From Wild Type and A350V IQSEC2 Mutant Mice

A single cell suspension from the mouse hippocampus was prepared by mechanical dissociation using the gentleMACS dissociator (Miltenyi Biotec, Gladbach, Germany) coupled with tissue enzymatic degradation using the Adult Brain Dissociation Kit (Miltenyi Biotec). The cell suspension was mesh-filtered (70 micron) to remove clumps and debris and red blood cells were removed by a Red Blood Cell Removal Solution (Miltenyi Biotec). A highly enriched population of neurons were obtained from this cell suspension by depleting non-neuronal cells using the Neuron Isolation Kit (Miltenyi Biotec). Non-neuronal cells are removed in this method using biotin-conjugated monoclonal antibodies specific for non-neuronal cells followed by anti-biotin monoclonal antibodies coupled to magnetic microbeads.

For flow cytometric analysis of membrane bound GluA1/2 we used the Alex Fluor 647 fluorochrome –conjugated to Anti-GluA1/2 antibody (Santa Cruz, sc-517265). This antibody recognizes an epitope present in both GluA1 and GluA2. Neurons were incubated with the antibody for 30 min at 4°C and were then washed with a phosphate-buffered staining solution (Dulbecco’s phosphate buffered saline with calcium, magnesium, glucose, pyruvate and 0.5% bovine serum albumin). Samples were analyzed on a LSRFortessa cell analyzer using FlowJo software.

### Surface Protein Cross-Linking Assay to Detect Surface AMPA Receptors in Hippocampal Tissue From Wild Type and A350V IQSEC2 Mice

To determine the relative distribution of surface AMPA receptors in the hippocampus of IQSEC2 A350V as compared to wild type IQSEC2 a surface protein-crosslinking assay was performed using membrane-impermeant crosslinking agent, Bis(sulphosuccinimidyl)suberate (BS_3_, Sigma) as previously described (Umanah et al., 2017) with some modifications. Bis(sulphosuccinimidyl)suberate (BS_3_) is a membrane-impermeant crosslinking agent that selectively crosslinks cell-surface proteins, forming high-molecular-mass aggregates. Non-crosslinked intracellular proteins still retain their normal molecular mass. Brains from wild type or IQSEC2 A350V mice littermates were rapidly removed and the hippocampi were dissected on ice and stored at -80°C until further processing. The frozen tissue was cut into small pieces. Each sample was divided into two and transferred to 1.5 ml Eppendorf tubes containing ice-cold PBS buffer with or without 2 mM BS_3_ followed by 3 h incubation at 4°C with gentle agitation. Tissues were quenched with 0.1 M glycine in PBS (10 min, 4°C) and lysed in ice-cold lysis buffer (PBS with 1% Triton-X100, 0.5% SDS, 5 mM ETDA, pH 7.4, and protease inhibitor cocktail). The lysates were homogenized and centrifuged at 15,000 × *g* for 5 min. The total protein concentrations in the supernatants were determined. 20 ug of total protein from each sample was resolved on 10% SDS-PAGE and western immunoblotting was performed to analyze the surface and intracellular pools of AMPA receptors using anti-GluA1 (rabbit monoclonal, Abcam, Ab109450) (1:1,000), anti-GluA2 (rabbit monoclonal, Abcam Ab150387) (1:2,000), anti-GluA3 (rabbit monoclonal, Abcam Ab40845) (1:1,000), anti-GluA4 (goat polyclonal, Abcam Ab115322) (1:1,000), HRP conjugated polyclonal goat anti-rabbit (Cell signaling 7074), HRP conjugated polyclonal rabbit anti-goat (Invitrogen 611620) and HRP conjugated monoclonal mouse anti-beta-actin (Millipore-SIGMA, A3854) (1:5,000). The signal on blots were generated with VisiGlo^TM^ HRP Chemiluminescent Substrate Kits (1B1583, AMRESCO) and imaged captured by Amersham Imager 600. The band intensities of all blots were measured using NIH ImageJ software (Rasband, W.S., NIH^2^). All experiments were performed with five biological replicates and quantitative data are presented as the mean ± standard error of the mean (SEM) performed by GraphPad prism6 software (Instat, GraphPad Software). Statistical significance was assessed by *t*-test (two-tailed). Assessments were considered significant with a *p* < 0.05.

### Immunocytochemistry of Hippocampus for Surface AMPA Receptor GluA2 From Wild Type and A350V Mice

Mice were anesthetized and transcardially perfused with 4% paraformaldehyde (PFA) in PBS after a brief vascular system washing with PBS as previously described (Umanah et al., 2017). After perfusion, brains were removed and postfixed overnight with 4% PFA plus 4% sucrose in PBS. Brains were paraffinized and sectioned. Brain sections were then deparaffinized and blocked with 5% normal goat serum for 1 h at room temperature (RT). To label surface GluA2, sections were incubated at 4°C overnight in PBS containing mouse monoclonal anti-N-terminal GluA2 Alexa 488- conjugated antibody (Millipore-SIGMA, MAB397A4). After four washes with PBS, sections were incubated with permeabilization buffer with 0.3% Triton X-100, 2.5% normal goat serum in PBS, and rabbit monoclonal anti-C-terminal GluA2 antibody (Abcam, Ab150387) to label total GluA2 for 4 h. The sections were then incubated in PBS containing goat anti-rabbit IgG Alexa Flour Plus 555 conjugated secondary antibody (Thermo Fisher Scientific, A32732) for 1 h at RT after four washes with PBS. Sections were then stained with DAPI for 5 min. After four washes with PBS, sections were mounted on precleaned slides with Immuno-Mount (Thermo Fisher Scientific). Images were acquired using a Zeiss LSM laser-scanning confocal microscope. Images for all conditions in individual experiments were analyzed by using identical acquisition parameters and were thresholded using identical values. The fluorescence intensities of labeled surface and internalized receptors were measured using ZEN software (Zeiss). Total and surface expression were normalized to the DAPI signal. Data are presented as mean ± SEM. The average fluorescence intensity of group results was used to determine the statistical significance by *t*-test (two-tailed) with a *p* < 0.05 being considered statistically significant.

### Electrophysiological Studies

#### Animals and Housing Conditions

Electrophysiological testing was performed at the Medical College of Wisconsin on A350V IQSEC2 and wild type IQSEC2 males at 18–20 weeks of age. Animals were housed 1–5 per cage in a 12 h light-dark cycle with food and water *ad libitum*. Experiments were conducted during the light phase.

#### Slice Preparation

Animals were anesthetized by isoflurane inhalation and decapitated. Coronal brain slices (360–400 μm thick) were cut using a vibrating slicer (Leica VT1200, Nussloch, Germany). Slices were prepared in a choline-based solution containing 110 mM choline chloride, 2.5 mM KCl, 1.25 mM NaH_2_PO_4_, 0.5 mM CaCl_2_, 7 mM MgSO_4_, 26 mM NaHCO_3_, 11 mM glucose, 11.6 mM sodium ascorbate, and 3.1 mM sodium pyruvate. Slices were cut in the midline to produce two individual slices from each section. The slices were incubated for 30 min in a sucrose-based solution containing 78 mM NaCl, 68 mM sucrose, 26 mM NaHCO_3_, 2.5 mM KCl, 1.25 mM NaH_2_PO_4_, 2 mM CaCl_2_, 2 mM MgCl_2_, and 25 mM glucose. Slices were then allowed to recover for at least 60 min in artificial cerebrospinal fluid (ACSF) containing 119 mM NaCl, 2.5 mM KCl, 4 mM CaCl_2_, 4 mM MgCl_2_, 1 mM NaH_2_PO_4_, 26 mM NaHCO_3_, and 11 mM glucose, at pH 7.4 and 290 mOsm. All solutions were saturated with carbogen (95% O_2_ and 5% CO_2_) at room temperature.

#### Input/Output Curve

Field potential evoked responses were recorded from the dendritic region of CA1 pyramidal neurons, with bipolar stimulation at the Schaffer collateral fibers, using Multiclamp 700A amplifier (Axon instruments). All recordings were made in circulating ACSF saturated with carbogen at 30°C. Fiber volleys and fEPSP (field excitatory post-synaptic potentials) slopes were calculated using Clampfit 10.7. Input/output (I/O) curves were generated from A350V IQSEC2 (*n* = 4) and wild type IQSEC2 (*n* = 5) male mice. One-tailed Student’s *t*-test was used to determine significance (*p* ≤ 0.05).

### Behavioral Tests

#### Animals and Housing Conditions

Behavioral testing was performed on A350V IQSEC2 and wild type (WT) IQSEC2 male and female mice on the same genetic background (C57BL/6J) at 5–7 weeks of age. Animals were housed in groups of 2–5 per cage in a reversed 12 h light-dark cycle (dim light at 9:30 am, lights off at 10:00 am) with food and water available *ad libitum*. The housing room was maintained at 23 ± 2°C. Experiments were conducted during the dark phase, under red lighting conditions (< 5 lux), between 10 am and 7 pm. All behavioral testing experiments were performed by the same two individuals who were blinded to the genotype of the animals. Any single animal was only handled by a single individual throughout these studies. Animals were handled for two consecutive days prior to the testing day except for the three-chamber sociability and social novelty tests (see below for details). On the testing day, mice were transferred to the testing room and were acclimated for an hour before the experiment commenced. All animal experiments were conducted in accordance with the United States Public Health Service’s Policy on Humane Care and Use of Laboratory Animals and approved by the Institutional Animal Care and Use Committee of Technion – Israel Institute of Technology (IL1691117).

#### Behavioral Analysis

For all the experiments, the arena or apparatus was cleaned after each trial with 70% ethanol and then with double-distilled water. All experiments were video-recorded by a camera (GUPPY PRO F-125B CCD) located above the arena and analyzed using Ethovision XT software version 10.1 (Noldus, Wageningen, The Netherlands), except for the Rotarod test which was recorded and analyzed using MATLAB R2017a (The Mathworks, Natick, MA, United States).

#### Open Field Test

5–6 week old mice were placed in the center of a squared box arena (40 × 40 × 35 cm) made of white Derlin plastic and explored the novel environment for 5 min (Prut and Belzung, 2003). Velocity and distance were measured to assess locomotor activity. Anxiety-like behavior was measured by calculating the time spent in the center as compared to the perimeter of the arena. The arena was divided into 25 equal squared tiles with the center of the arena defined as the nine central tiles (40% of the arena) with each corner defined as a single tile.

#### Rotarod

Assessment of motor coordination was done using the Rotarod test (Med Associates Inc., Georgia, VT, United States) (Karl et al., 2003). 5–6 week old mice were placed on a 32 mm diameter rod accelerating from 4 to 40 revolutions per minute (rpm) and latency to fall and end speed were measured. The test was performed on two consecutive days, each day consisting of four trials which lasted up to 6 min with an inter-stimulus interval of 10 min during which mice were placed in their home-cage.

#### Three-Chamber Sociability and Social Novelty Tests

Social interaction was measured in order to assess autistic-like behavior using a three-chamber test (Moy et al., 2004). Subject mice were assessed for the tendency to prefer an unfamiliar conspecific mouse (social stimulus; *Stranger 1*) over a novel object and over another unfamiliar mouse (novel social stimulus; *Stranger 2*). The arena (70 × 29 × 35 cm) was comprised of three chambers (side chambers 26 × 29 × 35 cm). 6–7 week old mice (subject and stimulus) were habituated to the testing room for 1 h on three consecutive days prior to the test day. Stimulus mice were further habituated to the wire cages (10.8 cm in height and 10.2 cm diameter; Galaxy Cup, Spectrum Diversified Designs, Inc., Streetsboro, OH, United States) for 20 min each day. On the test day, subject mice were habituated to the apparatus for 10 min and were allowed to explore all three empty chambers. Time spent in each chamber was measured to assess chamber bias. Following habituation, subject mice were assessed for social preference for 10 min by allowing interaction with Stranger 1 placed inside a wire cage in one chamber and a novel object placed inside an identical wire cage in the opposite chamber. Stimulus mice location was counterbalanced across trials to prevent chamber bias. Next, the novel object was replaced with Stranger 2 and social novelty was assessed for 10 min during which the subject mouse was able to choose between a novel mouse and an already familiar mouse. Time spent in each camber and in close interaction were measured for both the preference and novelty experiments. Stimulus mice were conspecific C57BL/6J mice from different litters, and were age, sex and weight-matched to the subject mice and to each other.

#### Morris Water Maze

The Morris water maze test was used to assess spatial learning and memory (Vorhees and Williams, 2006). 6–7 week old mice were placed in a 120 cm circular diameter pool filled with water and a 15 cm diameter transparent platform that was placed at the Southwest (SW) quadrant, submerged 1 cm below the water surface. Water was maintained at 22.0 ± 1°C and made opaque by adding a dried milk powder. Mice were trained for four consecutive days, each day consisted of four trials with an inter-stimulus interval of 15 min during which mice were placed in their home-cage. During training, mice were released from a different quadrant in each trial and were given 60 s to find the platform. If the mice did not find the platform within 60 s, the experimenter guided the animal to the platform. After reaching the platform, mice were left on the platform for 10 s. On the 5th day a probe trial was performed in which the platform was removed from the maze, mice were released at the Northeast quadrant and given 60 s to explore the maze. Time spent in the SW quadrant served as an index of long-term memory.

#### Statistical Analysis of Behavioral Tests

All data were analyzed using MATLAB R2017a (The Mathworks, Natick, MA, United States). Summary statistics are presented as means ± SEM. Lilliefors test was used to determine normality. Two-tailed Student’s *t*-tests were used on normally distributed data. Mann–Whitney *U*-test was used to analyze data when sample size was not sufficient to establish normality.

## Results

### In a Cell-Free System *in vitro* Wild Type IQSEC2 Binds Significantly Better to Apocalmodulin as Compared to A350V and Calcium Increases Binding of Both Wild Type and Mutant IQSEC2 to Calmodulin

As the A350V IQSEC2 mutation is in the IQ domain of IQSEC2 (Figure 1A) and the IQ domains of many proteins have been demonstrated to bind to calmodulin (Bahler and Rhoads, 2002) we first sought to determine how the A350V mutation would affect calmodulin binding to IQSEC2. Investigation of this interaction in a cell-free system *in vitro* using luciferase tagged IQSEC2 and calmodulin coupled to sepharose allowed for the precise control of calcium concentration which is known to dramatically affect the conformation and interactions of calmodulin with other proteins. In this system luciferase activity associated with calmodulin sepharose was used a quantitative readout of the calmodulin-IQSEC2 interaction. The IQ motif is present in over 100 proteins and in some cases preferentially binds to calcium-calmodulin while in other cases it binds preferentially to calcium-free calmodulin (apocalmodulin) (Bahler and Rhoads, 2002). Prior work on the IQSEC2-calmodulin interaction using myc-tagged wild type IQSEC2 and calmodulin sepharose (Myers et al., 2012) reported that apocalmodulin bound to IQSEC2 and that the addition of 2 mM calcium (thereby generating calcium-calmodulin) dramatically decreased the binding of calmodulin to IQSEC2. However, when we assessed the effect of calcium on the IQSEC2 calmodulin interaction we observed that calcium increased the binding of calmodulin to IQSEC2. Upon reviewing the buffers used by our group and that of Myers in assessing binding of IQSEC2 to calmodulin, we discovered that we differed in the concentration of Triton X-100 used in the binding and wash buffers. As shown in Figure 1B, at 0.1% Triton X-100, 100 uM calcium was associated with a several fold increase in binding of calmodulin to wild type IQSEC2 while at 0.5% Triton X-100, 100 μM calcium was associated with a several fold decrease in binding of calmodulin to wild type IQSEC2. Triton X-100 binds to most proteins via both hydrophobic and polar interactions (Singh and Kishore, 2006) and may thereby disrupt protein-protein interactions at high concentrations. An example of a calmodulin-interacting protein that is important for the calmodulin-IQ interaction is PEP-19 which binds to the C-terminal domain of calmodulin and electrostatically steers it to interact with the IQ domain (Wang and Putkey, 2016). We interpreted these results as indicating that normally calcium stimulates calmodulin binding to IQSEC2 and that the results of Myers showing calcium reduces the binding of calmodulin to IQSEC2 were artifacts related to the high Triton concentration used. Therefore, for our *in vitro* studies comparing the interaction between calmodulin and wild type IQSEC2 as compared to mutant IQSEC2 we used low (0.1%) Triton X-100. In the absence of added calcium (5 mM EGTA) we observed that apocalmodulin bound significantly better to wild type IQSEC2 than A350V IQSEC2 (146 ± 18 vs. 48 ± 8 luciferase units for wild type IQSEC2 as compared to A350V IQSEC2; *n* = 6 experiments, unpaired *t*-test, *p* < 0.001). We assessed the binding of wild type or A350V luciferase-IQSEC2 to calmodulin over a wide range of calcium concentrations. The Kd of calcium for calmodulin is 1 μM and it has been demonstrated that at free calcium concentrations of less than 200 nM all calmodulin exists as apocalmodulin and there is no calcium-calmodulin (Persechini and Cronk, 1999). As shown for a representative experiment in Figure 1C for both wild type and A350V IQSEC2, calcium resulted in an increase in the binding of calmodulin to IQSEC2 with a similar calcium dose-response relationship (half-maximal binding of calcium-calmodulin for IQSEC2 at a free calcium concentration of approximately 1 μM for both wild type and A350V IQSEC2). The binding of calcium calmodulin was significantly increased for A350V IQSEC2 as compared to wild type IQSEC2 at all calcium concentrations greater than 200 nM (A350V 594 ± 59.6 vs. wild type 271+/-41 luciferase units, *n* = 11, unpaired *t*-test, *p* < 0.0005). These data demonstrate that while A350V binds less efficiently than wild type IQSEC2 to apocalmodulin, the A350V mutant is capable of binding calcium-calmodulin equivalent to or even superior to wild type IQSEC2. As has been demonstrated for the binding of apocalmodulin and calcium calmodulin to myosin (Trybus et al., 2007) these data would suggest that the epitopes or conformations within IQSEC2 recognized by apocalmodulin and calcium-calmodulin are different.

### In Cells Wild Type IQSEC2 Binds More Effectively to Apocalmodulin Than A350V IQSEC2

**Figure 2:**
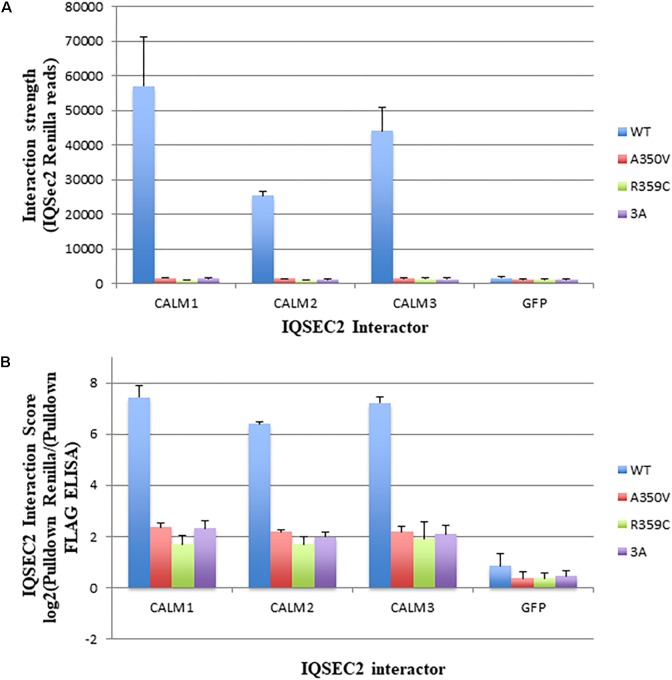
Binding of wild type and mutant IQSEC2 to calmodulins in cells using the Lumier assay. **(A)** Interaction strength of wild type and mutant IQSEC2 proteins and calmodulins, measured as renilla luciferase-IQSEC2 activity after pulldown of calmodulin-FLAG proteins. Shown are mean and SD of 4 biological replicate wells for each pair of IQSEC2 calmodulin interactions. **(B)** Interaction score of wild type and mutant IQSEC2 proteins and calmodulins, measured as log2 of renilla luciferase-IQSEC2 activity after FLAG pulldown divided by pulldown FLAG ELISA, to normalize for the interactor levels. Shown are the mean and SD of 4 biological replicate wells for each pair of IQSEC2 calmodulin interactions. Data comparing A350V IQSEC2 and wild type IQSEC2 are representative of four independent experiments done on independent days and the data for the R359C and 3a mutants are representative of two independent experiments done on independent days.

We next sought to determine if we could demonstrate differences in the binding of apocalmodulin to wild type and A350V IQSEC2 in cells similar to what we found in a cell free system. In order to achieve this goal we assessed the binding of wild type and three mutant IQSEC2 renilla luciferase constructs (A350V, R359C, 3A) to three isoforms of human calmodulin in HEK293T cells using the Lumier assay as described in methods. In HEK cells, the intracellular calcium concentration is 50–100 nM so that all calmodulin is present as apocalmodulin (Persechini and Cronk, 1999). We observed significantly stronger binding of wild type IQSEC2 to all three apocalmodulin proteins as compared to the three mutant IQSEC2 constructs. We observed 34, 15, and 36-fold differences between wild type and A350V IQSEC2 mutant with Calm1, Calm2 and Calm3, respectively (Figure 2A) with similar fold changes seen for IQSEC2 mutants R359C and 3A. Importantly, the interaction strength of the mutants with the apocalmodulins was very close to background interaction levels, as measured for interactions with GFP (Figure 2A) and GFP interaction was no different with wild type vs. mutant IQSEC2 (fold change of 1.07). Normalized interaction scores that take into account differences in the levels of the different interactors demonstrated 31, 14, and 31-fold higher interaction of wild type IQSEC2 with Calm1, Calm2 and Calm3, respectively, compared to A350V IQSEC2 (Figure 2B shown in log2 scale). We verified that the observed differences were not due to differences in cell viability or transfection efficiency as assessed by input luciferase activity of whole cell lysates. These data in cells are consistent with what was observed in a cell-free system, specifically that wild type IQSEC2 binds to apocalmodulin more effectively than A350V IQSEC2.

### Arf6 Activation Is Increased in A350V IQSEC2 Stable or Transiently Transfected Cells as Compared to Wild Type IQSEC2

**Figure 3:**
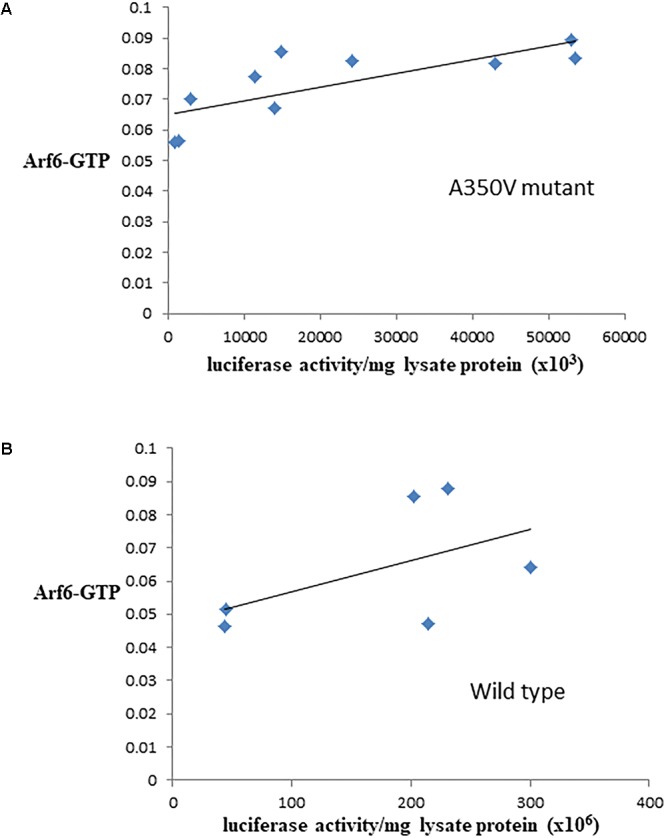
Correlation between Arf6-GTP and luciferase in cell lines producing A350V **(A)** or wild type **(B)** IQSEC2 luciferase. Arf6-GTP was measured by ELISA (spectrophotometric units). The correlation between the relative amount of IQSEC2 and Arf6-GTP was significant for A350V (*r* = 0.77, *n* = 10, *p* = 0.009) but not for WT IQSEC2 (*r* = 0.52, *n* = 6, *p* = 0.28).

The binding of calmodulin to IQSEC2 has been proposed to regulate IQSEC2 GEF activity for Arf6 promoting Arf6-GTP formation and thereby activating Arf6 (Shoubridge et al., 2010; Myers et al., 2012; Brown et al., 2016). Moreover, a R359C IQSEC2 mutation in the IQ calmodulin binding region of IQSEC2 has been associated with changes in IQSEC2 GEF activity for Arf6 (Shoubridge et al., 2010). Having demonstrated that the A350V affects the interaction of calmodulin for IQSEC2 we therefore set out to determine how the A350V mutation may affect the ability of IQSEC2 to promote Arf6-GTP. We assessed Arf6-GTP levels by ELISA in multiple stable transformants of HEK293T expressing different amounts of either wild type or A350V IQSEC2 as assessed by luciferase activity. We found, using equivalent amounts of protein extract, there was an approximately 25% increase in total Arf6-GTP in HEK293T cells expressing A350V IQSEC2 as compared to wild type IQSEC2 [0.075 ± 0.004 (*n* = 10) vs. 0.060 ± 0.015 (*n* = 6); *p* = 0.04]. The stable cell lines expressing wild type or A350V IQSEC2 differed markedly in the relative amount of IQSEC2 (assessed by luciferase activity) which they produced with overall 5–200 fold more wild type IQSEC2 being produced than mutant IQSEC2 in these cell lines. When normalized for both protein and luciferase activity (i.e., comparing the GEF specific activity of A350V IQSEC2 to wild type IQSEC2) the A350V IQSEC2 protein promoted nearly 30-fold more Arf6-GTP than wild type IQSEC2 protein (1.6 × 10^8^± 6.8 × 10^-9^ vs. 5.7 × 10^-10^± 1.7 × 10^-10^, *p* = 0.058). There was a highly significant correlation between the relative amount of A350V IQSEC2 and Arf6-GTP (*r* = 0.77, *p* = 0.009). This correlation was weaker in stable cell lines expressing different amounts of wild type IQSEC2 (*r* = 0.52, *p* = 0.28) (Figure 3A,B).

**Figure 4:**
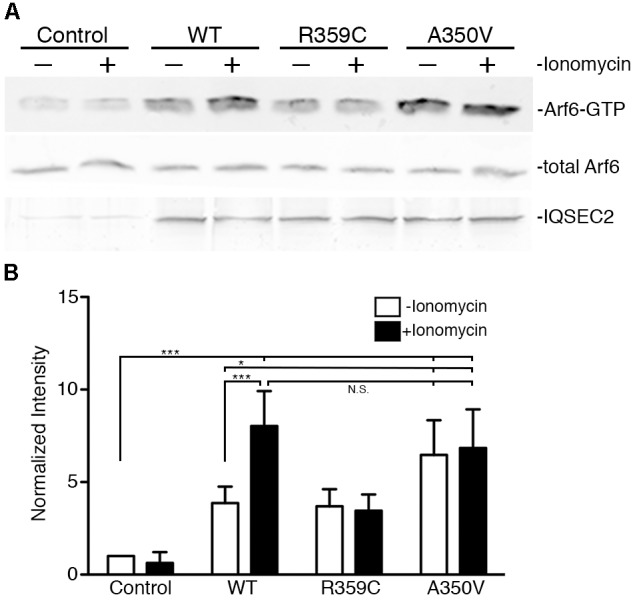
The A350V mutation activates ARF-GEF activity of IQSEC2. **(A)** WT, R359C, or A350V IQSEC2 was expressed in HEK293 cells, and treated with either ionomycin or carrier control. Activation of endogenous Arf6 under each condition was assayed in cell lysates by pull-down with GGA3. Precipitates were probed with antibodies against ARF6 (top row). Lysates were probed for total Arf6 expression and with antibodies against the FLAG tag on IQSEC2. **(B)** Arf6-GTP levels are calculated as fold increase over sham-transfected controls and are shown as mean ± SD from six independent experiments. The GGA pulldown assay was assessed by one-way ANOVA followed by Tukey’s Multiple Comparison Test for *post hoc* analysis. ^∗^*p* ≤ 0.01; ^∗∗∗^*p* ≤ 0.001; NS, Not significant.

We also assessed activation of Arf6 (Arf6-GTP) by wild type, A350V, or R359C IQSEC2 in a GGA3 pulldown assay. HEK 293T cells were transiently transfected with constructs to express either wild type, A350V or R359C IQSEC2, and endogenous Arf6 activation was tested with or without treatment with the calcium ionophore ionomycin. Ionomycin has been previously demonstrated to increase IQSEC2 GEF activity and Arf6-GTP formation by stimulating calcium influx and thereby affecting the interaction of calmodulin with IQSEC2 (Myers et al., 2012). Cell lysates were incubated with beads coated with GGA3:GST to isolate the GTP-bound Arfs, and bound Arf6 was assessed by immunoblot (Figure 4A). Expression of wild type IQSEC2 resulted in 3.87 ± 0.89 fold increase in Arf6 activation compared to sham transfected cells. Ionomycin treatment of cells containing wild type IQSEC2 further doubled Arf6 activation to an 8.03 ± 1.89 fold increase. Transfection with R359C also caused an induction of Arf6-GTP, but this mutant was unresponsive to ionomycin treatment. Transfection of the A350V mutant strongly increased Arf6 activation to a level that was statistically indistinguishable from the cells both transfected with wild type IQSEC2 and treated with ionomycin. In the absence of ionomycin transfection with A350V significantly increased Arf6 activation as compared to wild type (*p* < 0.01) and ionomycin did not further increase Arf6 activation by A350V (Figure 4B). These data demonstrate that the A350V mutation results in the constitutive activation of IQSEC2 GEF activity for Arf6.

### Surface AMPA Receptors Are Reduced in A350V Hippocampus

**Figure 5:**
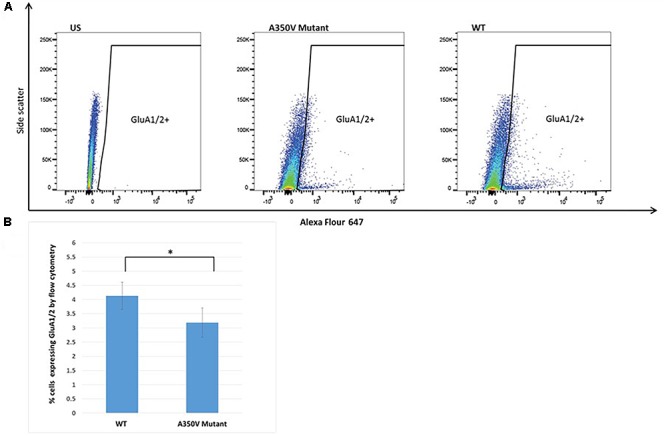
Flow cytometry analysis of hippocampal derived cells for GluA1/2. **(A)** Representative dot plot of one of 10 experiments. US panel-unstained hippocampal cells. Mut and WT panels demonstrating staining with Alexa Flour 647 for extracellular GluA1/2 in single cell suspension of hippocampal cells from A350V and wild type mice, respectively, and window selected for identifying GluA1/2+ cells. **(B)** Summary of all experiments demonstrating significantly higher percentage of cells staining with GluA1/2 in wild type as compared to A350V mutant hippocampal neuronal preparations (4.1 ± 0.5 vs. 3.1 ± 0.5, paired *t*-test, *n* = 10 independent experiments, ^∗^*p* < 0.00001).

**Figure 6:**
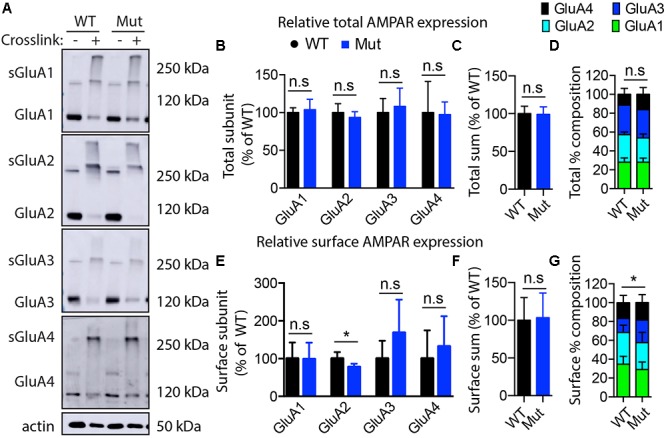
Assessment of AMPA receptor subunits by surface cross-linking. **(A)** Representative images of immunoblots after BS_3_-crosslinking of samples to assess surface (crosslinking “+”) and total protein expression of AMPA receptors (crosslinking “-”). Actin was used for normalization. **(B)** Quantification of relative total protein levels of AMPA receptor subunits in samples not crosslinked. Signal intensities normalized to actin signal and wild type as 100%. **(C)** Quantification of total sum of all AMPA receptor subunits in samples not crosslinked. **(D)** Quantification of AMPA receptor subunit percentage composition in samples not crosslinked. **(E)** Quantification of relative surface protein levels of AMPA receptor subunits in samples treated with BS_3_ (crosslinking “+”). Surface protein indicated by “s”on the blots in **(A)** Signal intensities of the bands at 250 kDa and above were normalized to actin signal and wild type as 100%. **(F)** Quantification of total sum of all AMPA receptor subunits at the membrane surface. **(G)** Quantification of surface AMPA receptor subunit percentage composition (significant differences in GluA2 composition). Optical densitometry quantification values represent the mean ± SEM (*n* = 4–5, ^∗^*p* < 0.05, n.s. *p* > 0.05, *t*-test, two-tailed).

**Figure 7:**
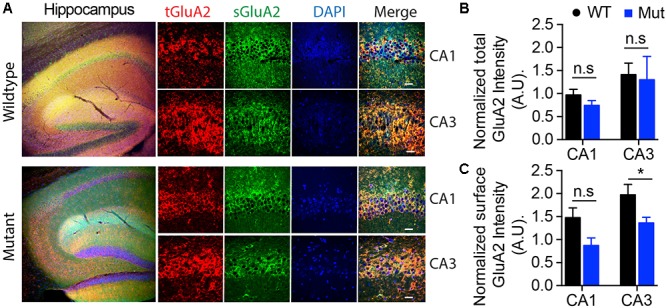
Assessment of surface expression of AMPA receptor GluA2 in hippocampus by immunocytochemistry. **(A)** Representative images of immunohistochemistry of total (tGluA2) and surface (sGluA2) AMPA receptor GluA2 in wildtype and A350V IQSEC2 mutant hippocampi. Higher power representative images of the CA1 and CA3 regions are shown (right panel). Labeled scale bar for high-resolution images = 20 μm. **(B)** Quantification of the normalized total GluA2 levels. Data represent mean ± SEM, *n* = 5 male mice in each group. No significant difference (n.s) *P* > 0.05, *t*-test, two-tailed. **(C)** Quantification of the normalized levels of surface GluA2 expression. Data represent mean ± SEM, *n* = 5 male mice in each group, ^∗^*p* < 0.05, n.s. *p* > 0.05, *t*-test, two-tailed.

The GEF activity of IQSEC2, mediated through Arf6, has recently been demonstrated to be required for the activity dependent removal of AMPA receptors from the surface of hippocampal neurons (Brown et al., 2016). We therefore proposed that a constitutive increase in Arf6-GTP by A350V, as we have demonstrated *in vitro*, would result in a down regulation of surface AMPA receptors in hippocampi from A350V IQSEC2 mice as compared to wild type IQSEC2 mice. We assessed expression of total gluA1 and gluA2 receptors in hippocampus and whole brain by western blot from wild type and A350V mice (age 6–8 weeks) and did not find any difference between the total receptor expression in these mice. We then sought to determine if the amount of surface expressed gluA1/2 was different between the wild type and A350V mice. First, by flow cytometry analysis (Figure 5A,B) we assessed gluA1/2 expression on the surface of neurons prepared from hippocampus as described in methods from 6 to 8 week old male mice and found a highly significant 30% reduction in the number of cells expressing surface gluA1/2 in A350V as compared to wild type IQSEC2 male mice (mean 4.1% ± 0.5% vs. 3.1% ± 0.5%,; median 3.9% vs. 2.7%, *n* = 10 for wild type and mutant mice, respectively, paired *t*-test, *p* < 0.00001.) There was also a highly significant reduction in the total amount of gluA1/2 surface expression in those cells identified by flow cytometry as expressing surface gluA1/2 (median difference 51, *p* < 0.008 by Wilcoxon signed rank test). Total (intracellular and extracellular) gluA1/2 was not significantly different between wild type and mutant mice assessed by flow cytometry. Second, using a surface cross linking assay coupled with western blot we assessed surface AMPA receptors (GluA1-4 subunits) in wild type and mutant hippocampi from male 6 to 8 week old mice and found that surface expression of GluA2 was significantly reduced in A350V IQSEC2 hippocampus as compared to wild type IQSEC2 hippocampus (Figure 6) resulting in an overall significant change in the surface distribution of the different AMPA receptor subunits in the A350V mice (unpaired *t*-test; Two-tailed, *t* = 2.579 *df* = 8, *p* = 0.0327). There was no significant difference between A350V and wild type hippocampus in the amount of total GluA2 AMPA receptor or in the distribution of the total AMPA receptor subunits in these studies. Additionally, immunohistochemistry of surface expression of GluA2 in hippocampus (Figure 7) further confirmed that GluA2 AMPA receptor surface expression is reduced in A350V male mice compare to control wild type male mice. There was no significant difference in the amount of total GluA2 between A350V and wild type hippocampi as assessed by immunohistochemistry. Collectively, these data demonstrate that hippocampal surface GluA2 is decreased in A350V IQSEC2 mice.

### Basal Synaptic Transmission Is Decreased in the Hippocampus of A350V IQSEC2 Mice

**Figure 8:**
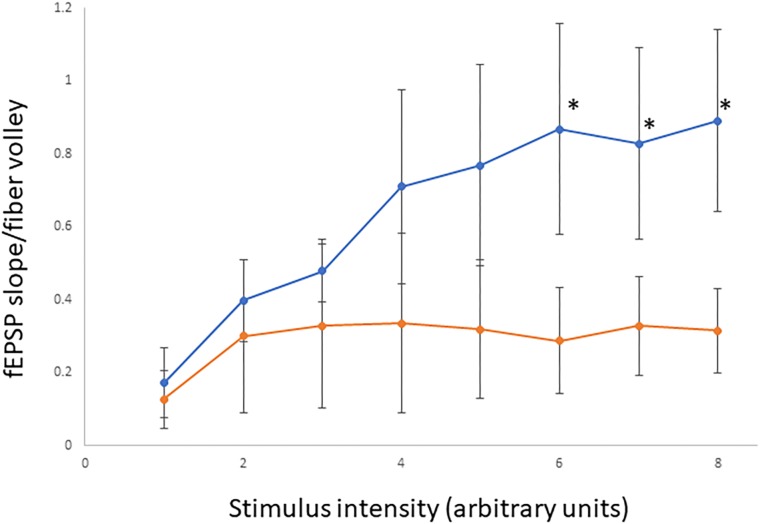
Assessment of basal synaptic transmission in CA1 region of hippocampus. Input/output curves representing synaptic responses (fEPSP slope/fiber volley) resulting from different stimulus intensities. Stimulus intensity numbers are arbitrary units; where 1 is intensity that gives minimum response and 8 is intensity that produces maximum response. Synaptic responses were significantly lower in A350V IQSEC2 mice (orange) compared to WT IQSEC2 mice (blue). Data represent mean ± SEM, *n* = 5 wild type IQSEC2 mice and *n* = 4 A350V IQSEC2 mice, ^∗^*p* ≤ 0.05, *t*-test, one-tailed.

In order to determine if the decrease in hippocampal surface AMPA expression was associated with a change in hippocampal synaptic transmission we performed electrophysiological testing using coronal brain slices from A350V and WT IQSEC2 mice as described in methods. Evoked responses were recorded from the dendritic region of hippocampal CA1 pyramidal neurons. An input/output curve representing synaptic responses (fEPSP slope/fiber volley) resulting from different stimulus intensities (Figure 8). Synaptic responses were significantly lower in A350V IQSEC2 mice as compared to WT IQSEC2 mice. These data demonstrate that basal hippocampal synaptic transmission is decreased in A350V IQSEC2 mice.

### Behavioral Phenotyping of A350V IQSEC2 Mice

**Figure 9:**
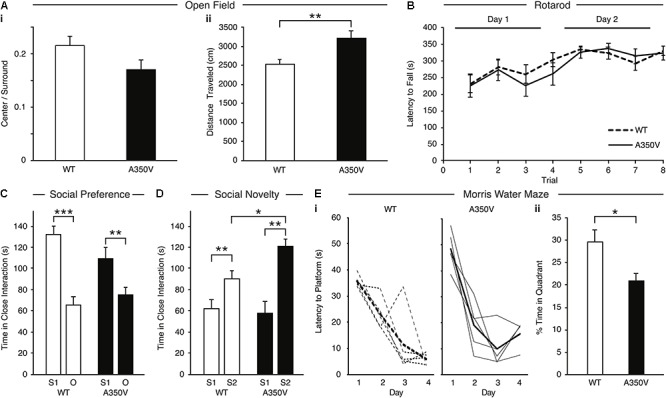
Behavioral phenotype of A350V IQSEC2 mouse model. **(A)** A350V display normal levels of anxiety-like behavior (i) and increased locomotion activity in an open field test (ii) (*n* = 13 wild type and A350V IQSEC2 mice). **(B)** Intact motor coordination in A350V relative to WT tested by the Rotarod test expressed as an equivalent learning curve and retention across two days in the two groups (*n* = 13 wild type and A350V IQSEC2 mice). **(C)** Duration of time spent in close interaction with Stranger 1 (S1) and an object (O) in the three-chamber social preference test demonstrating both A350V and WT preferred interacting with S1 although this preference was less pronounced in A350V (*n* = 13 wild type and A350V IQSEC2 mice). **(D)** Preference for social novelty expressed as the duration of time spent in close interaction with a previously encountered stranger animal (S1) relative to a novel stranger animal (S2) revealed a tendency to spend more time with S2 in WT and A350V, with significant enhancement of this preference for social novelty in A350V mice (*n* = 12 wild type and 9 A350V IQSEC2 mice). **(E)** Learning and memory deficits in A350V mice shown by the latency to reach the hidden platform during training (i), shown for individual animals (*thin lines*) and averaged across the group (*thick line*) separately for WT and A350V mice and the percentage time spent in the target quadrant in a Morris water maze test (ii) (*n* = 5 wild type and A350V IQSEC2 mice). Data indicate means ± SEM. ^∗^*p* < 0.05, ^∗∗^*p* < 0.01, ^∗∗∗^*p* < 0.001.

In order to characterize the behavioral phenotype of the A350V IQSEC2 animal model, we examined anxiety-like behavior, locomotion, motor coordination, social behavior and learning abilities. Anxiety-like behavior was assessed in the open field test and we found no significant difference between A350V male mice (*n* = 13) and WT littermates (*n* = 13) in the time spent in the center of the arena relative to the perimeter [A350V: mean = 0.215+/-0.02; WT: mean = 0.17+/-0.01, unpaired *t*-test; *t*(24) = 1.588, *p* = 0.130], suggesting that A350V show normal levels of anxiety-like behavior (Figure 9Ai). Locomotion (total distance traveled) was also assessed using the open field test (Figure 9Aii). There was a significant difference between A350V (*n* = 13) and WT littermates (*n* = 13) male mice when measuring the total distance traveled and velocity in the open field arena [unpaired *t*-test; distance: *t*(24) = -3.2805, *p* < 0.01; velocity: *t*(24) = -3.2851, *p* < 0.01]. This result was consistent with the increased locomotion found in A350V mice in the habituation phase of the three-chamber social preference test [unpaired *t*-test; *t*(24) = 2.3125, *p* < 0.05]. Motor coordination was assessed in the Rotarod test (Figure 9B). Both A350V and WT mice performed at similar levels with no significant difference between the two groups in the time spent on the accelerating rotating rod. Social preference of A350V mice toward an unfamiliar conspecific mouse over an inanimate novel object was assessed using a three-chamber social arena (Figure 9C). Both A350V male mice (*n* = 13) and WT (*n* = 13) littermates preferred to spend more time in close interaction with a social stimulus (Stranger 1) over a novel object [paired *t*-test; A350V: *t*(12) = 2.535, *p* < 0.05; WT: *t*(12) = 4.671, *p* < 0.001]. However, A350V mice showed a trend toward social impairment as measured by a decreased time spent in close interaction with Stranger 1 compared to WT mice [unpaired *t*-test; *t*(24) = 1.743, *p* = 0.094]. Preference for social novelty (Figure 9D) was assessed by comparing the time spent in close interaction with a novel mouse to an already familiar mouse (Stranger 1). Both A350V (*n* = 9) and WT (*n* = 12) mice preferred an unfamiliar mouse (Stranger 2) relative to a previously encountered mouse (paired *t*-test; A350V: *t*(8) = 4.367, *p* < 0.01; WT: *t*(11) = 3.134, *p* < 0.01). However, A350V mice spent significantly more time in close interaction with a novel social stimulus (Stranger 2) over the familiar mouse compared to WT mice [unpaired *t*-test; *t*(19) = 2.294, *p* < 0.05]. Hippocampal-dependent memory was assessed using the Morris water maze test. Significant differences between female A350V (*n* = 5) and WT (*n* = 5) mice were found in the latency to reach the platform on the 1st and 4th days of training (Mann–Whitney *U*-test; Day 1: *U* = 16, *p* < 0.05; Day 4: *U* = 16, *p* < 0.05), as shown in the learning curve of each group (Figure 9Ei). In addition, time in the target quadrant during a probe trial was compared between female A350V (*n* = 5) and WT (*n* = 5) mice revealing a significant difference between the groups (Mann–Whitney *U*-test; *U* = 39.5, *p* < 0.05) (Figure 9Eii). Collectively, these data demonstrate that the A350V IQSEC2 mice model manifests some of the abnormalities found in the human index case with the A350V IQSEC2 mutation, specifically hyperactivity, abnormal social interactions and impaired cognitive function.

## Discussion

This study provides new findings for understanding the regulation of IQSEC2 activity and the pathophysiology of a new IQSEC2 mutant (A350V) with implications for drug therapy. First, we have demonstrated that calcium increases the binding of IQSEC2 to calmodulin and we reconcile our data with previously reported conflicting results. Second, we report on the first mutation identified in humans associated with a constitutive activation of Arf6 due to a constitutive increase in IQSEC2 GEF activity. Third, we have demonstrated that surface GluA2 AMPA receptors are decreased in the brains of A350V IQSEC2 mice. Finally, we demonstrate that A350V IQSEC2 mice have abnormal behavioral phenotypes with increased locomotion, abnormal social interactions and decreased learning.

We have demonstrated in two different systems, *in vitro* with cell extracts and in cells, that apocalmodulin can bind to IQSEC2 and that this binding is impaired with the A350V mutant. In a resting cell (i.e., HEK 293T cells, neurons) the cytoplasmic concentration of free calcium is 50–100 nM (Persechini and Cronk, 1999) with localized calcium concentrations of 1–10 μM being achieved with an appropriate stimulus (i.e., NMDA receptor activation in neurons). The K_d_ of calcium for calmodulin is approximately 1 μM with essentially no calcium calmodulin being found in a cell with a free cytoplasmic calcium of less than 200 nM (Persechini and Cronk, 1999). Our demonstration that half-maximal interaction between calmodulin and IQSEC2 occurs at around 1 μM calcium is therefore physiologically relevant.

However, while A350V binds less efficiently to apocalmodulin as compared to wild type IQSEC2, the A350V mutant is capable of binding calcium-calmodulin equivalent to or even superior to wild type IQSEC2. Similar to myosin (Trybus et al., 2007) the epitope or conformation within the IQ region of IQSEC2 recognized by apocalmodulin and calcium calmodulin may be different. Our finding that A350V IQSEC2 can effectively bind to calcium-calmodulin but not apocalmodulin may suggest that the alpha helical distortion of the IQ domain in the A350V IQSEC2 mutant induced by the valine-for-alanine substitution introduces a change in the conformation of the IQ motif of IQSEC2 that is similar to the conformation of the IQ motif that is induced in wild type IQSEC2 by the binding of calcium calmodulin. The wild type IQSEC2 IQ motif may adopt a relaxed conformation in which apocalmodulin may bind when calcium-calmodulin is not present; however, in the A350V mutation the conformation of the IQ region may be locked in a conformation which will not allow it to assume a conformation permissive for apocalmodulin binding.

This is the first demonstration of a human disease resulting from a constitutive activation of Arf6 due to a constitutive increase in IQSEC2 GEF activity. We have shown that the IQSEC2 GEF activity for Arf6 is increased in cells expressing mutant A350V IQSEC2 as compared to wild type IQSEC2. Ionomycin treatment of cells expressing WT IQSEC2 induced a significant increase in Arf6 activation, indicating a calcium-dependent regulation of Arf-GEF activity. The A350V mutant, however, already had a high level of basal activity that was comparable to WT IQSEC2 after treatment with ionomycin. All previously reported mutants of IQSEC2, including R359C, which is also located in the IQ region, have been noted to have decreased Arf6 GEF activity (Shoubridge et al., 2010). We also show that the GEF activity of the R359C mutant is not elevated by ionomycin treatment, which suggests that deficits in calcium dependent regulation of this mutant may contribute to ID. As discussed above we propose that the constitutive activation of IQSEC2 GEF activity by the A350V mutation may be due to the mutation locking the IQ motif into the same conformation as wild type IQSEC2 bound to calcium-calmodulin (wherein IQSEC2 GEF activity is increased).

A key factor underlying the strength of individual excitatory synapses is the number of AMPA receptors at synapses. Trafficking of AMPA receptors to and from synapses plays a key role in synaptic transmission and in experience-dependent synaptic plasticity and associative learning (Qin et al., 2005; Rumpel et al., 2005; McCormack et al., 2006; Hu et al., 2007; Matsu et al., 2008; Kielland et al., 2009; Zhu, 2009). NMDA receptor-induced removal of GluA1/2 AMPA receptors from synapses is a key step in the induction of long-term depression (LTD), and Arf6 activation is a necessary component of this type of plasticity (Scholz et al., 2010; Brown et al., 2016). IQSEC2 regulates AMPA receptor currents (Myers et al., 2012; Brown et al., 2016). The Arf-GEF activity of IQSEC2 is required for LTD, as ID-linked mutations in IQSEC2 that decrease its Arf-GEF activity impair its induction (Brown et al., 2016). This indicates that properly regulated activation of Arf6 by IQSEC2 is necessary for normal synaptic plasticity processes, including the regulated removal of AMPA receptors in LTD. Our findings here showing that increased Arf6 activity is associated with decreased GluA2 AMPA surface expression in A350V mutant brain tissue is consistent with the critical role of Arf6 in regulating the removal of AMPA receptors from the plasma membrane (Brown et al., 2016). Our demonstration that basal hippocampal synaptic transmission is decreased in A350V IQSEC2 mice is also consistent with a down-regulation of AMPA receptors in this model. Future experiments with specific AMPA receptor antagonists and positive allosteric modulators will attempt to prove that the decreased synaptic transmission in the A350V mice is due at least in part to a downregulation of AMPA receptors.

**Figure 10:**
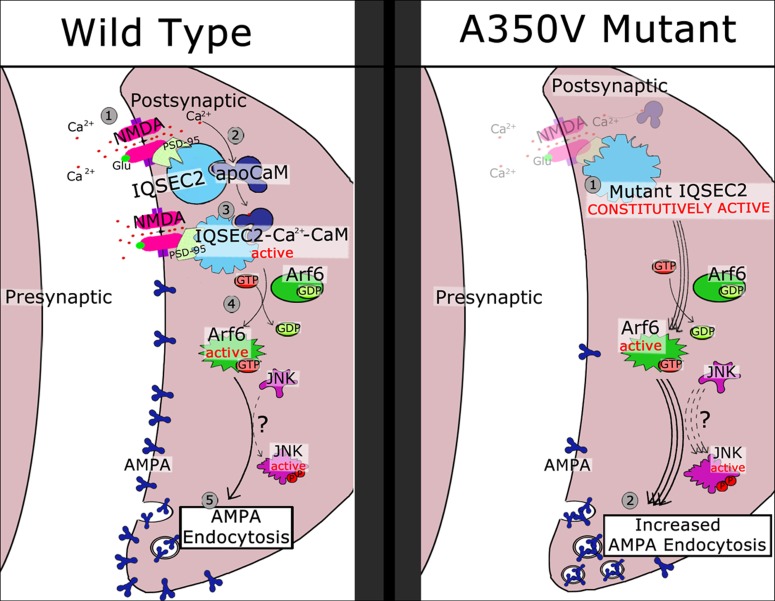
Schematic model of regulatory cascade mediated by wild type and A350V IQSEC2 mutant and consequences for AMPA trafficking. Wild type IQSEC2. (1) Activation of NMDA receptor with calcium influx. (2) Calcium can bind to apocalmodulin which is already bound to IQSEC2. (3) Ca-calmodulin bound to IQSEC2 induces a conformational change in IQSEC2 that leads to activation of IQSEC2 GEF activity (4) resulting in formation of active Arf6-GTP. Arf6-GTP promotes endocytosis of surface AMPA receptors (5) by pathways that are poorly understood, but which may include JNK (Myers et al., 2012). A350V mutant IQSEC2. (1) A350V IQSEC2 has conformation similar to active form of wild type IQSEC2 and has constitutive GEF activity capable of promoting increased Arf6-GTP formation (2). Increased Arf6-GTP results in more pronounced down regulation of surface AMPA receptors (3).

Based on the data presented here and previous work presented by others (Myers et al., 2012; Brown et al., 2016) we propose in Figure 10 a model for the activation of wild type IQSEC2 by calcium as well as the pathophysiological consequences of the A350V IQSEC2 mutation. In the presence of wild type IQSEC2, the binding of glutamate to the NMDA receptor leads to calcium influx and a rise in free intracellular calcium. This calcium binds to calmodulin and the binding of calcium calmodulin to the IQ site on IQSEC2 induces the GEF activity of IQSEC2 allowing it to promote the formation of Arf6-GTP. Arf6-GTP, in turn, regulates endocytosis of surface AMPA receptors by pathways that are poorly understood, but which may include JNK (Myers et al., 2012). On the other hand, in the A350V IQSEC2 mutant, IQSEC2 GEF activity for Arf6 is constitutively activated resulting in persistently increased Arf6 activity, which may markedly downregulate surface AMPA receptors, specifically GluA2, (representing an exaggerated form of long-term depression). Thus, the normal processes for regulating AMPA receptor levels at synapses are compromised by the A350V IQSEC2 mutation. This hypothesis appears to provide a mechanistic basis for the defects in behavior and learning associated with the A350V IQSEC2 mutation. Furthermore, according to this hypothesis, treatment to restore the balance in AMPA transmission by blocking exaggerated AMPA downregulation may provide clinical benefit specifically an increase in learning potential. The size of the change in surface GluA2 AMPA induced by the A350V mutation is similar to what has been reported in mutations in the Thorase gene (Umanah et al., 2017; Piard et al., 2018) where pharmacological attempts to restore normal surface AMPA activity have shown therapeutic benefit in man (Ahrens-Nicklaus et al., 2017).

Behavioral phenotyping of the A350V IQSEC2 mice demonstrates increased locomotion, abnormal social interactions and learning impairments in the absence of motor coordination deficits. The increased preference for social novelty in the A350V mice described here, while different from what has been described in autism, has been described in other genetic encephalopathies with intellectual disability and abnormal social functioning such as Williams’s syndrome (Martin et al., 2018; Ng et al., 2018). Our behavioral findings in the A350V mice appear to model some of the abnormal behaviors found in the human index case with the A350V mutation, specifically hyperactivity, abnormal social interactions with no inhibitions with strangers and impaired cognitive function. However, more complete behavioral phenotyping of the A350V IQSEC2 model will be required in order to properly define the spectrum of social interaction abnormalities, hyperactivity features, and the type of learning and memory impairment present in these mice. Decreases in surface AMPA receptors have been noted in other models of learning impairment (neurodevelopmental as well as Alzheimer’s disease) and in models of social dysfunction (Guntupalli et al., 2016; Ahrens-Nicklaus et al., 2017; Umanah et al., 2017; Kim et al., 2018; Piard et al., 2018; Tian et al., 2018) and increasing AMPA transmission has shown benefit in these models (Lauterborn et al., 2016; Kim et al., 2018). Accordingly, strategies designed to restore surface AMPA in the A350V IQSEC2 mouse model may have a beneficial effect on cognitive and affective behavior and represents a potential actionable node for treatment in humans for the A350V IQSEC2 mutation.

## Ethics Statement

This study was carried out in accordance with the recommendations of the Technion Faculty of Medicine and the Medical College of Wisconsin Institutional Animal Care and Use Committees (IACUC) and approved by the IACUC committees.

## Author Contributions

ER, RJ, KS-R, MS, MF, NL, RM, RW, RS, RP, DL, AK, IK, MC, NG, GU, and AL designed and performed the experiments, and analyzed the data. RS, RW, DL, NG, IK, GU, and AL wrote the manuscript. All authors were involved in revising the manuscript for important intellectual content and gave final approval of the version to be published.

## Conflict of Interest Statement

The authors declare that the research was conducted in the absence of any commercial or financial relationships that could be construed as a potential conflict of interest.

## Footnotes

1 https://somapp.ucdmc.ucdavis.edu/pharmacology/bers/maxchelator/CaEGTA-TS.htm

2 http://rsb.info.nih.gov/ij/
